# Unraveling salivary microbiota diversity following kidney transplantation: insights from baseline peripheral blood lymphocyte subsets

**DOI:** 10.1080/20002297.2025.2490284

**Published:** 2025-04-08

**Authors:** Xuyu Xiang, Tianyin Wang, Peng Ding, Yi Zhu, Ke Cheng, Yingzi Ming

**Affiliations:** a The Transplantation Center of the Third Xiangya Hospital, Central South University, Changsha, China; b Engineering and Technology Research Center for Transplantation Medicine of National Health Commission, Changsha, China

**Keywords:** Biomarker, immune monitoring, salivary microbiota, peripheral blood lymphocyte subsets, kidney transplantation

## Abstract

**Background:**

Effective biomarkers are urgently needed to monitor immune suppression in kidney transplantation (KT) recipients. Our study identified a close association between the salivary microbiota and immunosuppressant concentrations. It is therefore hypothesized that the salivary microbiota may be linked to immune function.

**Materials and Methods:**

We analyzed 108 saliva samples from 37 KT patients using 16S rRNA sequencing. Patients were clustered via K-means based on peripheral blood lymphocyte subset (PBLS) counts.

**Results:**

Cluster1 exhibited significantly higher CD4^+^ T cells (*p* < 0.0001), CD8^+^ T cells (*p* < 0.0001), and B cells (*p* = 0.0071) versus Cluster2, with marginally NK cells (*p* = 0.2319). Beta diversity indicated significant differences in microbial communities. LEfSe analysis identified 34 differential taxa at the genus level. A random forest model in a fivefold three-times repeated cross-validation, developed with differential taxa, discriminated patient groups well (AUC, 75.61% ± 14.54%), with Pseudopropionibacterium most contributing. Meanwhile, only Pseudopropionibacterium correlated with more than 2 PBLSs. Cluster2 was predicted to exhibit more primary and secondary bile acid synthesis, with differential expression of related enzymes.

**Conclusion:**

The absolute count of PBLSs is correlated with the composition of the salivary microbiota, with the strongest association observed between Pseudopropionibacterium and lymphocytes. Our study provides novel insights into immune monitoring post-KT.

## Introduction

Kidney transplantation (KT) stands as a pivotal intervention for end-stage renal disease (ESRD) patients, enhancing survival rates and alleviating economic burdens. Postoperative immunosuppression is the cornerstone of daily management for KT recipients. However, excessive immunosuppression predisposes individuals to fatal infections and drug toxicities, while insufficient immunosuppression heightens the risk of rejection [1]. Effective monitoring of immune function is essential to optimize immunosuppressive therapy, yet current methods often require invasive procedures and are not always reliable in predicting post-transplant outcomes.

In recent decades, various effective biomarkers for immune monitoring have emerged. Measurement of blood drug concentrations of immunosuppressants, which affect the proliferation and activity of lymphocytes, is currently the most widely used clinical method [2]. Additionally, immune-related effector molecules [3–5], distribution of immune cells and subsets, cell-based immunity assays [6] and replication dynamics of latent viruses [7] have been found to effectively reflect patients’ immune function. Among these, our center has discovered that the absolute counts of peripheral blood lymphocyte subsets (PBLSs) could predict the occurrence and progression of post-kidney transplant infections [8,9]. Moreover, The dynamic changes of lymphocyte subpopulations have been found to correlate with rejection [10–12]. Lymphocytes play a critical role in immune response following KT. However, due to limitations such as inadequate efficacy, technical difficulties, and high manpower and resource costs, there is still a lack of effective, non-invasive, and feasible tools in clinical practice.

Our previous research revealed a profound shift in the salivary microbiota of KT patients, which was strongly associated with renal function recovery [13,14]. Furthermore, notable distinctions were observed among patients with varying tacrolimus trough concentrations [15]. These findings push us to hypothesize the potential link between salivary microbiota and the immune system, particularly lymphocytes.

Thus, we aim to investigate the relationship between PBLSs and salivary microbiota diversity and composition. By identifying microbial taxa associated with PBLSs, this study seeks to establish salivary microbiota as a novel, non-invasive biomarker for immune monitoring in KT recipients, potentially improving patient management and outcomes.

## Materials and method

### Study design

This was a retrospective study to evaluate the association between the baseline PBLSs and salivary microbiota during the perioperative period. Both inpatients and outpatients underwent KT from 1 October 2022 to 1 April 2023 in the Transplantation Center, The Third Xiangya Hospital, Central South University. The exclusion criteria included [1] without PBLSs test before KT [2]; age less than 18 years old or more than 65 years old [3]; multiple-organ transplantation. All patients who were not excluded were enrolled.

### Immune monitoring panel for PBLSs

The BD Multitest 6-color TBNK reagent with BD Trucount tube which identified the percentages and absolute counts of total lymphocytes (TBNK, namely CD3^+^ T cells, CD19^+^ B cells, and CD16^+^/CD56^+^ NK cells), CD3^+^ T cells (a total of CD3^+^CD4^+^ T cells and CD3^+^CD8^+^ T cells), CD3^+^CD4^+^ T cells, CD3^+^CD8^+^ T cells, CD19^+^ B cells and CD16^+^/CD56^+^ NK cells was used for PBLS test. This panel was performed according to the manufacturer’s instructions and analyzed by BD FACSCanto clinical software (BD BioSciences, San Jose, CA, USA). Briefly, 50 μL fresh whole blood from EDTA anticoagulation tube was used for detection. Cells and TBNK reagent were incubated in the Trucount tube for 15 min in the dark. After erythrolysis, samples were detected using BD FACSCanto II.

### Unsupervised clustering

We performed data analysis for baseline absolute count of PBLSs with the K-means clustering algorithm to classify different health states into k clusters given n objects, by R package ‘cluster’ (2.1.6). The variables, absolute count of CD4^+^ T cells, CD8^+^ T cells, B cells, and NK cells, were utilized for clustering, resulting in the division of all participants into two groups.

### Sample collection

Saliva samples were collected at various time points after surgery, including 1 day, 3 days, 7 days and 14 days for all patients. Before collection, patients fasted for half an hour and rinsed their mouths. Saliva samples were collected between 3 and 5 pm, and no stimulation was applied to the patients during sample collection. Patients spit the saliva into a sterile tube until it reaches 2 ml. Saliva was stored at −80°C immediately after collection.

### 16S rRNA sequencing and data analysis

The details of 16S rRNA sequencing were consistent with our previous study [13]. Briefly, DNA was extracted using Magnetic Soil and Stool DNA Kit (TIANGEN). After quality and concentration tests, the V3-V4 hypervariable regions of bacterial 16S ribosomal gene were quantified by quantitative PCR (qPCR) using the specific primer 341F (CCTAYGG-GRBGCASCAG) and 806 R (GGACTACNNGGGTATCTAAT) with the barcode. Sequencing libraries were generated using NEBNext® Ultra^TM^ DNA Library Prep Kit for Illumina (NEB, USA) following the manufacturer’s recommendations, and sequenced by the Illumina NovaSeq 6000 platform.

Paired-end reads from the original DNA fragments were merged using FLASH. Sequences were clustered into amplicon sequence variants (ASVs) by DADA2 in QIIME2 [16] and then each ASV was annotated based on Silva 138 database. Further analysis and visualization were performed by the R package ‘MicrobiotaProcess’ (1.13.2.994) [17]. Six indexes, Observe, Chaos1, Ace, Shannon, Simpson, and Pielou, were used to evaluate alpha diversity. We used Bray-Curtis distance-based principal co-ordinates analysis (PCoA) to visualize the microbiota composition and permutational multivariate analysis of variance (ADONIS) to assess whether there was significant variation in beta diversity among groups. Linear discriminant analysis Effect Size (LEfSe) was used for the quantitative analysis of biomarkers within different groups, and a value of Linear Discriminant Analysis (LDA) >2 was required for results to be considered statistically significant.

Furthermore, abundance of functional pathways and enzyme were predicted using the software PICRUSt2 [18], with subsequent data analysis and visualization carried out using the R package ‘ggPICRUSt2’ (1.7.3) [19]. The Benjamini-Hochberg method was employed to adjust p-values. A significance threshold of less than 0.001 was applied for functional pathways, while for enzymes, it was set at less than 0.0001.

### Model building

The classification ability of the salivary taxa for patients with different baseline PBLSs was analyzed through random forest algorithm by R package ‘caret’ (6.0–94) [20] and ‘random forest’ (4.7–1.1) [21], and receiver operating characteristic (ROC) curves was visualized through R package ‘pROC’ (1.18.4). All significant variants in Alpha diversity and LEfSe analysis were included for model building, and the random forest algorithm model was used to build the classifier in a fivefold three-times repeated cross-validation. The area under the curve (AUC), accuracy, recall and F1 score were used to evaluate the ability of the classifier, and the mean accuracy decrease and mean gini decrease were used to evaluate the contribution of variants to the classification ability of the model.

### Statistical analysis

All statistical analyses were performed using R (4.2.1), SPSS software 26 (IBM, Chicago, IL, USA), and GraphPad Prism 8 (GraphPad Software, America). A unpaired Wilcoxon rank-sum test, unpaired t-test, chi-square test, Fisher’s exact test and one-sample Wilcoxon test were used appropriately to evaluate the significance of differences in data between groups. Speraman’s test was used appropriately to analyze the correlation. A *p* < 0.05 was required for results to be considered statistically significant.

## Results

### Unsupervised clustering and general characteristics of study population

**Figure 1.**
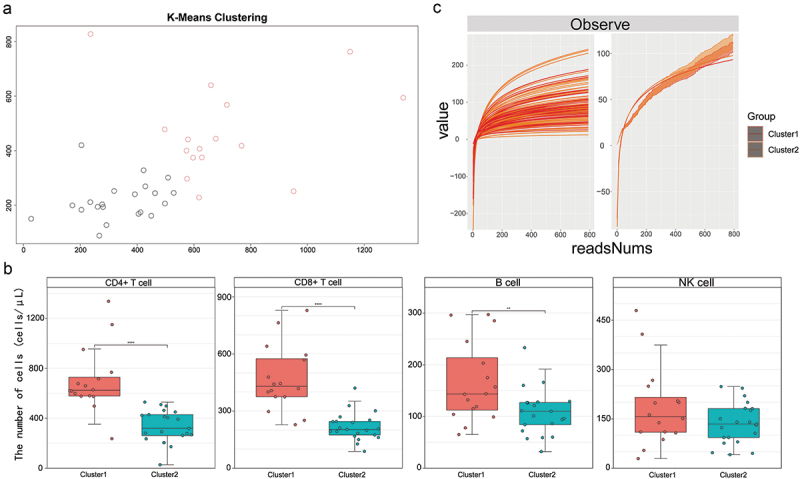
KT recipients were divided into 2 clusters by K-means clustering algorithm. (a) Scatter plot of K-means clustering based on cluster features; (b) distribution of PBLSs absolute counts in two groups; (c) line graph demonstrated the effect of read numbers on the Observe index in sample and group. KT, kidney transplantation; PBLSs, peripheral blood lymphocyte subpopulations.

As there is currently no clear cutoff value to distinguish different immune statuses, we incorporated information on baseline absolute lymphocyte counts (including CD4^+^ T cells, CD8^+^ T cells, B cells, and NK cells), and utilized unsupervised K-means clustering algorithm to classify all patients into two clusters, designated as Cluster1 and Cluster2 (Figure 1(a)), mean individual silhouette widths = 0.3875).

A total of 37 patients with 108 saliva samples were included in the study, with 16 patients classified into Cluster1. Demographic and clinical characteristics of the two groups are summarized in Table 1. Significant differences were observed between the two groups in terms of the absolute count of CD4^+^ T cells (*p* < 0.0001), CD8^+^ T cells (*p* < 0.0001), B cells (*p* = 0.0071). Although the NK cell count was also higher in Cluster1, the difference did not reach statistical significance (*p* = 0.2319, Figure 1(b)). Other variables showed no differences between the two groups, including serum creatinine (Scr) levels, immunosuppressant blood concentrations and sample distribution. Additionally, Figure 1(c) illustrates the observed index across samples and groups at various read numbers, with most sample curves reaching a plateau. This observation validates the adequacy of the read numbers utilized in our study.

**Table 1.** General characteristics of KT recipients with different level of baseline PBLS.

		Cluster 1 (*n* = 16)	Cluster 2 (*n* = 21)	p-value
The Cell Number of PBLSs (cells/μL), mean ± SD				
CD4+ T cells		699.00 ± 259.40	335.60 ± 130.20	<0.0001
CD8+ T cells		469.10 ± 170.40	217.00 ± 72.84	<0.0001
NK cells		184.50 ± 121.00	135.70 ± 62.40	0.2319
B cells		166.10 ± 77.14	108.50 ± 44.49	0.0071
Distribution of sample, n (%)				0.8915
1 day		10 (62.50%)	17 (80.95%)	
3 days		12 (75.00%)	14 (66.67%)	
7 days		14 (87.50%)	17 (80.95%)	
14 days		11 (68.75%)	13 (61.90%)	
Gender, n (%)				0.0501
Male		15 (93.75%)	13 (61.90%)	
Female		1 (6.25%)	8 (38.10%)	
Age (years), mean ± SD		41.31 ± 12.00	44.71 ± 9.66	0.3987
Body Mass Index (kg/m^2^), mean ± SD		22.62 ± 3.96	22.79 ± 3.50	1.0000
Induction Therapy, n (%)				0.7727
ATG		14 (87.50%)	19 (90.48%)	
Non-ATG		2 (12.50%)	2 (9.52%)	
Antibiotics, n (%)				0.1395
Beta-lactam		16 (100.00%)	21 (100.00%)	
Engpolymyxin		1 (6.25%)	6 (28.57%)	
Glycopeptides		1 (6.25%)	0 (0.00%)	
Anti-fungal drugs		7 (33.33%)	4 (19.05%%)	
Dialysis Type, n (%)				0.2873
HD		10 (62.50%)	11 (52.38%)	
PD		6 (37.50%)	7 (33.33%)	
None		0 (0.00%)	3 (14.29%)	
Dialysis Duration (months), mean ± SD		22.63 ± 16.23	21.94 ± 21.28	0.6512
Cause of End Stage Renal Disease, n (%)				1.0000
IgA Nephropathy		1 (6.25%)	1 (4.76%)	
Unknown		15 (93.75%)	20 (95.24%)	
Scr before KT (μmol/L), mean ± SD		1063.00 ± 271.40	1032.00 ± 348.60	0.5493
Scr after KT (μmol/L), mean ± SD				
7 days		201.70 ± 133.60	331.10 ± 432.70	0.7331
14 days		116.10 ± 33.74	262.10 ± 393.60	0.5588
C_0_ of Tacrolimus after KT (μmol/L), mean ± SD				
7 days		7.19 ± 2.53	8.41 ± 3.73	0.4900
14 days		9.69 ± 2.48	8.33 ± 2.28	0.1791

KT, kidney transplantation; PBLS, peripheral blood lymphocyte subpopulations; SD, standard deviation; ATG, antihuman thymocyte globulin; HD, hemodialysis; PD, peritoneal dialysis; Scr, serum creatinine; C0, trough blood concentrations.

### The baseline peripheral blood lymphocyte subpopulations was significantly associated with the salivary microbiota composition

**Figure 2.**
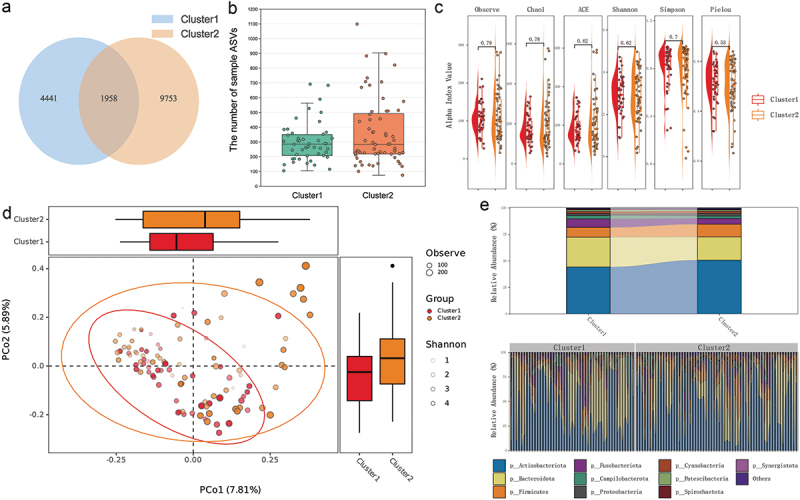
The overall structure and diversity of salivary microbiota between Cluster1 and Cluster2. (a) Venn graph for the group ASVs; (b) bar plot for the number of sample ASVs; (c) violin plot for alpha diversity between the 2 groups; (d) PCoA graph of salivary microbiota composition, and also shown the degree of explanation of PCs, the distribution over the PCs, and the alpha diversity indices, observe and Shannon, for each sample; (e) top 10 salivary species composition of samples and groups at phylum level. ASV, amplicon sequence variant; PCoA, principal co-ordinates analysis.

Next, the overall structure and diversity of salivary microbiota between the two groups were compared. The number of ASVs distributed in Cluster 2 was nearly double that of Cluster 1, with less than 12.12% of ASVs shared between the groups (Figure 2a). However, at the individual level, there was no significant difference in the number of ASVs (Figure 2b). Similarly, alpha diversity, including indexes Observe, Chao1, Ace, Shannon, Simpson and Pielou, did not show significant inter-group differences (Figure 2(c)). Regarding beta diversity, ADONIS analysis revealed significant differences between the two groups (*p-value* = 0.0031, Figure 2(d)). Furthermore, the combined relative abundance of *Actinobacteriota*, *Bacteroidota* and *Firmicutes* exceeded 75% in both groups, and there was no differences in the abundance of the individual top 10 taxa at the Phylum level (Figure 2(e)).

### The certain taxa linked with absolute count of peripheral blood lymphocyte subpopulations

**Figure 3.**
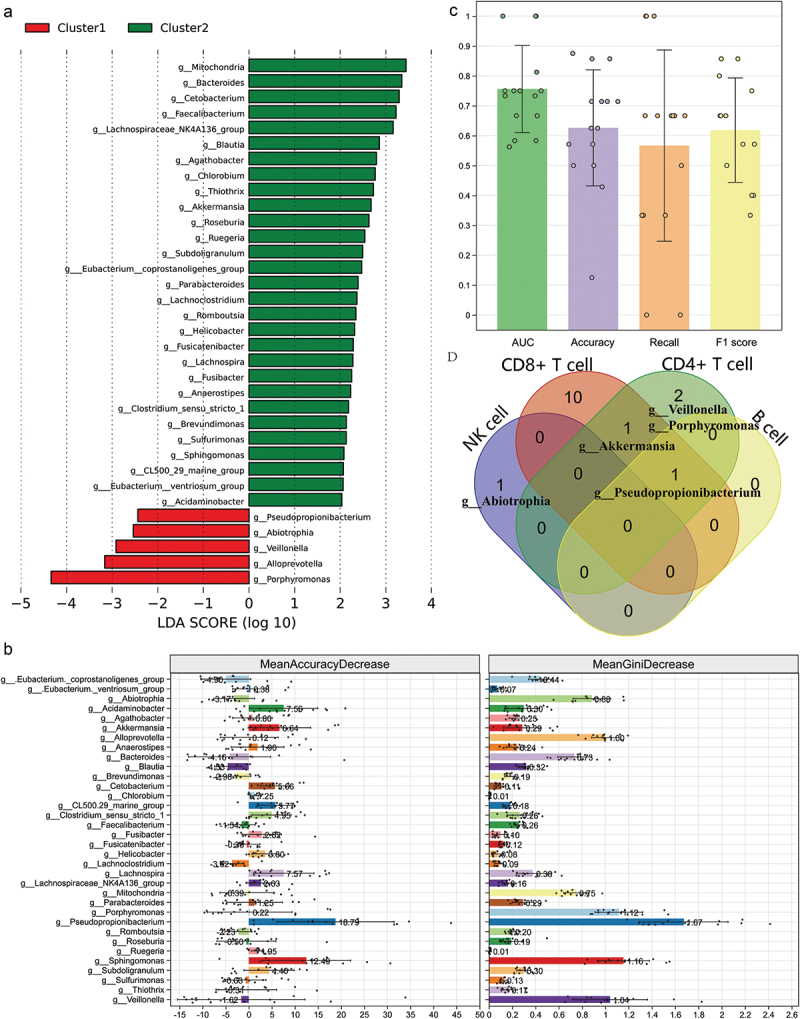
Differential taxa could effectively distinguish patients with different levels of PBLSs and were significantly associated with the absolute count. (a) The LDA of significant taxa in LEfSe analysis; (b) the importance of variants in random forest model; (c) bar plot for the performance of random forest model; (d) the overlap of taxa significantly associated with each lymphocyte count. PBLSs, peripheral blood lymphocyte subpopulations; LDA, linear discriminant analysis; LEfSe, linear discriminant analysis effect size.

The relationship between certain taxa and lymphocyte counts was further analyzed. LEfSe analysis identified 34 differential biomarkers at the genus level, with *Porphyromonas* being the most prominent enriched taxa in Cluster1, while *Mitochondria* was predominant in Cluster2 (Figure 3(a)). Initially, these biomarkers were indiscriminately used to establish a random forest model in a fivefold three-times repeated cross-validation. Variable importance, both the mean accuracy decrease and mean gini decrease, revealed that *Pseudopropionibacterium* contributed the most to classification ability (Figure 3(b)). The classifier effectively distinguished between the two patient groups (Figure 3(c); AUC, 75.61% ± 14.54%, one-sample Wilcoxon test *p-value* <0.0001; Accuracy, 62.62% ± 19.42%; Recall rate, 56.67% ± 32.00%; F1 score, 61.85% ± 17.47%), albeit with some variability across folds (especially the indexes accuracy and recall rate). Furthermore, the correlation between biomarkers and absolute counts of each lymphocyte subtype was explored by Speraman’s test (Table S1), along with the overlap of taxa (Figure 3(d)). Results indicated a highest abundance of taxa associated with CD8^+^ T cells, while *Pseudopropionibacterium*, which contributed most to the classifier, also showed correlations with three indicators CD4^+^ T cells, CD8^+^ T cells and B cells.

## Differential functional pathways and enzyme between Cluster1 and Cluster2

To understand the underlying mechanisms of these associations, the functional pathways and enzymes abundance was predicted based on the salivary microbiota, and differences in their distributions between the two clusters were analyzed. In terms of functional pathways, both primary bile acid biosynthesis and secondary bile acid biosynthesis were enriched in Cluster2 (Supplemental Fig. S1a and b). ADONIS analysis revealed significant differences in the distribution of functional abundances between the two groups (Supplemental Fig. S1c; *p-value* = 0.0498). Among the differential enzymes, four enzymes directly related to bile acid metabolism were identified, including 4-hydroxybutyrate dehydrogenase, 4-hydroxybutanoyl-CoA dehydratase, Vinylacetyl-CoA Delta-isomerase and Alanine-glyoxylate transaminase (Supplemental Figure S1D and 1E). Additionally, there may be overall changes in the distribution of enzymes (Supplemental Figure S1F; *p-value* = 0.0608).

## Discussion

In this retrospective study, a total of 39 patients, with 108 post-operative saliva samples, were ultimately included. Although several studies have highlighted the close association between pre-transplant PBLSs and various post-kidney transplantation outcomes [8,22–24], there is currently no clear cutoff value to distinguish different immune statuses. Therefore an unsupervised K-means algorithm based on the absolute counts of PBLSs was employed to cluster KT recipients. Consequently, 16 patients were assigned to Cluster1, while the remaining 21 patients were assigned to Cluster2. Comparison between Cluster1 and Cluster2 revealed significantly higher counts of CD4^+^ T cells, CD8^+^ T cells, and B cells in Cluster1. Although the NK cell count was also higher in Cluster 1, the difference did not reach statistical significance. As Scr and sample distribution was similar between 2 groups, that the renal function drives alterations in salivary microbiota during perioperative period [13,14], time points were combined for analysis.

Subsequent analysis showed that while individual ASVs, alpha diversity, and the composition of the top 10 phyla were similar between the two groups, beta diversity revealed significant differences in overall salivary microbiota composition. Unique ASVs represented over 69.4% of each group’s microbiota. Further investigation into the relationship between microbial taxa and PBLSs identified 34 differential taxa using LEfSe analysis. These taxa were used to build a random forest model, which effectively distinguished the two patient groups (AUC: 75.61% ± 14.54%, *p* < 0.0001), with *Pseudopropionibacterium* contributing most significantly. Spearman analysis revealed that only *Pseudopropionibacterium* abundance was positively correlated with multiple lymphocyte subgroups (CD4^+^ T cells, CD8^+^ T cells, and B cells). Functional pathway predictions indicated higher synthesis of primary and secondary bile acids in Cluster2 compared to Cluster1, with four bile acid metabolism-related enzymes showing differential expression between the groups.

Despite recipients of KT undergoing induction and maintenance therapy, along with intense immune reconstitution processes [25], pre-transplant PBLSs continue to sequentially influence post-transplant immune function. Hélène Longuet et al. found baseline CD4^+^ T cell count is a significant independent predictor for long-term impaired CD4^+^ T-cell reconstitution [26]. A study from our center demonstrated that the absolute number of baseline CD4^+^, and CD8^+^ T cells, as well as B cells and NK cells, were notably reduced in the patients developed pneumonia within 1 year after KT compared to the stable group [8]. Moreover, pre-transplant NKG2C^+^ NK cells bearing adaptive markers were specifically associated with a reduced incidence of post-transplant symptomatic cytomegalovirus infection [22]. In addition to infection, PBLSs are also associated with tumor occurrence and acute rejection, that cancer was associated with a decreased pre-transplant proportion of CD4^+^CD45RChigh T cells, and the increase of frequency of CD8^+^CD45RChigh T cells was associated with acute rejection [24]. Therefore, we believe that exploring the composition of salivary microbiota based on PBLSs absolute count levels will further our understanding of the close association between salivary microbiota and post-kidney transplantation immune function.

In our study, we found significant differences in the beta diversity of salivary microbiota among patients with different baseline lymphocyte counts. Alterations in diversity and overall structure were also noted in the salivary microbiota of HIV patients with immune statuses similar to KT recipients. Compared to HIV-negative individuals, HIV-positive patients exhibit higher alpha diversity and significantly different beta diversity in their salivary microbiota [27]. This difference remained consistent regardless of HIV progression [28], while antiretroviral therapy reduces alpha diversity and shifts beta diversity closer to that of healthy controls [27]. However, the impact of treatment effectiveness on the overall composition of salivary microbiota remains contradictory. Studies by Yirui Xie et al. found no diversity differences between immunological responders and non-responders [29], while Rachel M Presti et al. observed significantly higher bacterial richness and diversity in samples obtained from participants with sustained lower CD4^+^ T cell counts after 24 weeks of treatment [30]. Furthermore, salivary microbiota can influence the distribution of immune cells. Exposure to the oral microbiota postnatally recruits neutrophils to the neonatal epithelium through γδT17 cells [31]. Additionally, periodontal bacteria can infiltrate the epithelium, activate signaling pathways, induce inflammation, and block natural killer and cytotoxic cells, all of which contribute to the carcinogenesis cycle [32].

Given the significant contribution of *Pseudopropionibacterium* to the classifier and its positive correlation with lymphocyte subgroup counts, it is considered closely associated with the immune function of KT recipients. However, research on *Pseudopropionibacterium* remains limited. *Pseudopropionibacterium* is a novel gram-positive, pleomorphic, rod-shaped, non-spore forming, non-motile organism isolated from human oral cavity [33]. Compared to common dental plaque, *Pseudopropionibacterium* is a major component of black stain [34]. A higher abundance of *Pseudopropionibacterium* has also been observed in the saliva of children with extrinsic black tooth stain [35]. Moreover, its abundant presence was noted in matched frozen specimens from the apical region of patients with apical periodontitis [36], with even higher levels seen in asymptomatic patients [37]. *Pseudopropionibacterium* depletion is distinctive in the oral-pharyngeal microbiota of COVID-19 patients compared to individuals with influenza B infection and healthy controls [38]. It is also an effective microbial biomarker for monitoring recovery in COVID-19 patients [39]. Further investigation is warranted to elucidate the causal relationship between *Pseudopropionibacterium* and lymphocytes.

The enrichment of *g_mitochondria* in Cluster2 is another intriguing finding. Mitochondrial DNA in saliva primarily originates from salivary gland epithelial cells and leukocytes [40]. The increased abundance of this DNA may suggest mitochondrial dysfunction in these cells. Mitochondrial dysfunction in salivary glands, including impaired oxidative phosphorylation and excessive reactive oxygen species (ROS) production, leads to the release of mitochondrial DNA and ROS [41]. These molecules can activate innate immune pathways, such as the STING pathway, resulting in local inflammation [42]. The inflammatory microenvironment promotes the migration and infiltration of peripheral blood lymphocytes into salivary gland tissue [42]. In patients with Sjögren’s syndrome, the degree of lymphocyte infiltration in the salivary glands correlates positively with markers of mitochondrial damage [43], which may contribute to a reduced peripheral lymphocyte count. Additionally, mitochondrial dysfunction in lymphocytes may alter their energy metabolism [41], accelerating apoptosis and further promoting the release of mitochondrial DNA into saliva [44], thereby reducing lymphocyte numbers.

Consistent with the comparison of predicted functional pathways and enzyme abundances in this study, primary and secondary bile acids emerge as critical mediators linking the digestive tract microbiota to lymphocytes. The microbiota serves as a crucial regulator of host bile acid metabolism. For example, Pu-erh tea inhibits microbes associated with bile salt hydrolase activity, leading to increased levels of ileal conjugated bile acids [45]. Repurposing disulfiram suppresses secondary bile acid biosynthesis by reducing 7α-dehydroxylation mediated by *Clostridium*, thus ameliorating non-alcoholic steatohepatitis [46]. Altered bile acid metabolism can impact immune cell phenotype and function either through direct stimulation [47] or by modulating Farnesoid-x-receptor activity [45,48], facilitating chemical communication with the host immune system.

Recent studies have confirmed the existence of the microbiota-bile acid-immune cell axis [49–51]. The conversion of primary bile acids mediated by the gut microbiota regulates changes in hepatic sinusoidal endothelial cell CXCL16 expression, influencing the accumulation of hepatic CXCR6^+^ NKT cells [52]. In Treg-deficient mice, antibiotic treatment restored levels of several primary and secondary bile acids, significantly reducing IL-6 expression in vitro in RAW macrophages induced with inflammation, thereby offering substantial protection [53]. Additionally, gut microbiota play a crucial role in the onset and progression of inflammatory bowel disease. *Bacteroides uniformis* regulates the mucosal layer by participating in bile acid metabolism and modulating key metabolites (α-muricholic acid, chenodeoxycholic acid, and lithocholic acid), thus inhibiting TH17 cell differentiation [54]. Alterations in gut microbiota induced by metabolic dysfunction-associated steatotic liver disease lead to increased secondary bile acid levels in the ileum. In cases of impaired intestinal barrier function, this results in severe CD8^+^ T cell-mediated ileitis [55].

However, the PICRUSt software infers microbial functions based on its database, without considering the influence of specific environments or symbiotic microorganisms on microbial activity. Therefore, precise data still require further validation by metabolomic sequencing and whole-genome sequencing. If microbiota-derived metabolites are confirmed to interact with pathways involved in the immunosuppression of KT recipients, more accurate immune monitoring and effective intervention strategies will significantly benefit patient prognosis.

Tacrolimus may serve as a key link between the salivary microbiota and lymphocytes. As a primary immunosuppressant that inhibits lymphocyte proliferation and activity, its pharmacokinetics in CYP3A5 non-expressing KT recipients have been shown to correlate with both gut microbiome diversity and the specific bacterial species [56]. The abundance of *Faecalibacterium prausnitzii* in stool samples 1 week post-transplant can predict the tacrolimus dose required to achieve the desired serum concentration 1 month later [57]. In mouse models, gut microbiome depletion affects ABCB1 expression in intestinal tissues, influencing tacrolimus pharmacokinetics [58]. Our previous research also identified a correlation between tacrolimus trough levels and salivary microbiome diversity in KT recipients, with *Capnocytophaga* showing a negative correlation with drug levels at both the population and individual levels [15]. Therefore, the salivary microbiome may colonize the gut, influencing tacrolimus pharmacokinetics and modulating its immunosuppressive effects on lymphocytes.

There were several limitations in this study. The sample size is small, and there is a lack of external data for validation. Furthermore, experiments to prove the conclusions and further investigate the mechanisms are lacking. However, these findings further bolster our confidence in salivary microbiota as potential biomarkers of immune suppression. Therefore, our center will increase the sample size, extend the follow-up period, and further explore the role of salivary microbiota in improving the prognosis of KT recipients.

The absence of periodontal examinations represents another significant limitation of our study. Numerous studies have reported a close relationship between salivary microbiome composition, oral hygiene, and periodontal health [59–63]. Certain salivary microbial features have been shown to reflect the severity of periodontitis [64,65], with pathogens such as *Porphyromonas gingivalis* from the red complex identified as key contributors to its development [66]. In summary, oral health is strongly correlated with the salivary microbiota, and understanding a patient’s periodontal condition may improve the reliability of outcomes. Therefore, we will consider incorporating periodontal health status in future research.

## Conclusion

In conclusion, this study identifies a specific and significant correlation between baseline PBLSs and the salivary microbiota composition in KT recipients. Notably, the strongest correlation was observed for *Pseudopropionibacterium*, suggesting that this taxon may play a unique role in modulating immune function in this population. These findings highlight the potential of *Pseudopropionibacterium* as a non-invasive biomarker for immune monitoring post-KT, offering new avenues for research and clinical application. Further studies are needed to elucidate the underlying mechanisms of this association, and to explore the broader implications of *Pseudopropionibacterium* in transplant immunology.

## Supplementary Material

Supplemental materials.docx

## Data Availability

16S rRNA Sequencing data are deposited under SRA PRJNA904953 and PRJNA963071.
