# Supplementary material for: Unraveling salivary microbiota diversity following kidney transplantation: insights from baseline peripheral blood lymphocyte subsets
Source: J Oral Microbiol. 2025 Apr 8;17(1):2490284. doi: 10.1080/20002297.2025.2490284 (PMC11983535; doi:10.1080/20002297.2025.2490284)
Supplement: Supplemental materials.docx [file ZJOM_A_2490284_SM9716.docx]

Supplemental Fig. 1. The correlation between primary and secondary bile acid synthesis and PBLSs levels. (A) The differences in functional pathways between the two groups; (B) Heatmap of functional pathway abundance; (C) PCoA graph of functional pathways composition; (D) The differences in enzymes between the two groups; (E) Heatmap of enzyme abundance; (F) PCoA graph of enzymes composition. PBLSs, peripheral blood lymphocyte subpopulations; PCoA, principal co-ordinates analysis.

| Table S1. The relationship between relative abundance of biomarker and absolute count of peripheral blood lymphocyte subpopulations | | | | | | | | | | | | | | | |
| --- | --- | --- | --- | --- | --- | --- | --- | --- | --- | --- | --- | --- | --- | --- | --- |
|  | CD4+ T cell | | |  | CD8+ T cell | | |  | NK cell | | |  | B cell | | |
|  | Spearman rho | 95% confidence interval | P value |  | Spearman rho | 95% confidence interval | P value |  | Spearman rho | 95% confidence interval | P value |  | Spearman rho | 95% confidence interval | P value |
| g__.Eubacterium._coprostanoligenes_group | -0.1883 | -0.4905 to 0.1542 | 0.2643 |  | -0.225 | -0.5190 to 0.1166 | 0.1806 |  | 0.04872 | -0.2889 to 0.3755 | 0.7746 |  | 0.03926 | -0.2975 to 0.3673 | 0.8175 |
| g__.Eubacterium._ventriosum_group | -0.06847 | -0.3924 to 0.2706 | 0.6872 |  | -0.418 | -0.6592 to -0.09893 | 0.01 |  | -0.006256 | -0.3384 to 0.3273 | 0.9707 |  | 0.06765 | -0.2713 to 0.3917 | 0.6908 |
| g__Abiotrophia | 0.05427 | -0.2837 to 0.3803 | 0.7497 |  | 0.06123 | -0.2773 to 0.3862 | 0.7188 |  | 0.3386 | 0.006442 to 0.6035 | 0.0404 |  | 0.1413 | -0.2010 to 0.4529 | 0.4042 |
| g__Acidaminobacter | -0.01283 | -0.3442 to 0.3214 | 0.9399 |  | -0.2528 | -0.5402 to 0.08741 | 0.1311 |  | 0.3091 | -0.02656 to 0.5821 | 0.0627 |  | 0.05502 | -0.2831 to 0.3809 | 0.7464 |
| g__Agathobacter | -0.02282 | -0.3530 to 0.3124 | 0.8934 |  | -0.2488 | -0.5372 to 0.09167 | 0.1375 |  | -0.05852 | -0.3839 to 0.2798 | 0.7308 |  | -0.04793 | -0.3748 to 0.2896 | 0.7782 |
| g__Akkermansia | -0.331 | -0.5980 to 0.002160 | 0.0454 |  | -0.5209 | -0.7276 to -0.2275 | 0.0009 |  | -0.1476 | -0.4580 to 0.1948 | 0.3832 |  | -0.2521 | -0.5397 to 0.08823 | 0.1323 |
| g__Alloprevotella | 0.265 | -0.07450 to 0.5494 | 0.113 |  | 0.3061 | -0.02986 to 0.5799 | 0.0654 |  | -0.03059 | -0.3598 to 0.3054 | 0.8574 |  | 0.25 | -0.09039 to 0.5381 | 0.1356 |
| g__Anaerostipes | -0.1312 | -0.4446 to 0.2109 | 0.439 |  | -0.2469 | -0.5357 to 0.09373 | 0.1408 |  | 0.02014 | -0.3149 to 0.3507 | 0.9058 |  | -0.1432 | -0.4544 to 0.1992 | 0.3979 |
| g__Bacteroides | -0.2156 | -0.5118 to 0.1263 | 0.1999 |  | -0.3249 | -0.5936 to 0.008918 | 0.0497 |  | -0.1542 | -0.4633 to 0.1884 | 0.3622 |  | -0.01891 | -0.3496 to 0.3160 | 0.9115 |
| g__Blautia | -0.1093 | -0.4266 to 0.2321 | 0.5197 |  | -0.235 | -0.5267 to 0.1062 | 0.1615 |  | -0.1161 | -0.4323 to 0.2255 | 0.4936 |  | -0.06074 | -0.3858 to 0.2778 | 0.721 |
| g__Brevundimonas | -0.06388 | -0.3885 to 0.2748 | 0.7072 |  | -0.1742 | -0.4793 to 0.1685 | 0.3025 |  | 0.2178 | -0.1240 to 0.5135 | 0.1953 |  | -0.02167 | -0.3520 to 0.3135 | 0.8987 |
| g__Cetobacterium | -0.1992 | -0.4990 to 0.1432 | 0.2373 |  | -0.3812 | -0.6337 to -0.05532 | 0.0199 |  | -0.3046 | -0.5788 to 0.03152 | 0.0668 |  | -0.03424 | -0.3630 to 0.3021 | 0.8406 |
| g__Chlorobium | -0.1724 | -0.4779 to 0.1703 | 0.3076 |  | -0.3672 | -0.6238 to -0.03908 | 0.0254 |  | -0.1166 | -0.4327 to 0.2251 | 0.4921 |  | -0.1181 | -0.4339 to 0.2236 | 0.4864 |
| g__CL500.29_marine_group | -0.1522 | -0.4617 to 0.1903 | 0.3686 |  | -0.1131 | -0.4298 to 0.2284 | 0.5052 |  | 0.04533 | -0.2920 to 0.3726 | 0.7899 |  | 0.01849 | -0.3163 to 0.3492 | 0.9135 |
| g__Clostridium_sensu_stricto_1 | -0.2155 | -0.5116 to 0.1265 | 0.2003 |  | -0.4845 | -0.7039 to -0.1808 | 0.0024 |  | -0.2606 | -0.5461 to 0.07917 | 0.1193 |  | -0.1913 | -0.4928 to 0.1512 | 0.2567 |
| g__Faecalibacterium | -0.08505 | -0.4064 to 0.2551 | 0.6167 |  | -0.2997 | -0.5752 to 0.03681 | 0.0715 |  | -0.1248 | -0.4395 to 0.2171 | 0.4617 |  | -0.08474 | -0.4062 to 0.2553 | 0.618 |
| g__Fusibacter | -0.2692 | -0.5525 to 0.06999 | 0.1072 |  | -0.5243 | -0.7298 to -0.2319 | 0.0009 |  | -0.2235 | -0.5179 to 0.1181 | 0.1835 |  | -0.1545 | -0.4636 to 0.1880 | 0.3611 |
| g__Fusicatenibacter | -0.1634 | -0.4707 to 0.1792 | 0.3338 |  | -0.307 | -0.5806 to 0.02881 | 0.0646 |  | -0.07888 | -0.4012 to 0.2609 | 0.6426 |  | -0.09913 | -0.4182 to 0.2417 | 0.5594 |
| g__Helicobacter | -0.2448 | -0.5341 to 0.09595 | 0.1443 |  | -0.3845 | -0.6360 to -0.05916 | 0.0188 |  | -0.2611 | -0.5465 to 0.07862 | 0.1186 |  | -0.2179 | -0.5136 to 0.1240 | 0.1951 |
| g__Lachnoclostridium | -0.1363 | -0.4488 to 0.2060 | 0.4213 |  | -0.2605 | -0.5461 to 0.07923 | 0.1194 |  | -0.1075 | -0.4252 to 0.2338 | 0.5266 |  | -0.1411 | -0.4527 to 0.2012 | 0.4048 |
| g__Lachnospira | -0.2048 | -0.5034 to 0.1375 | 0.224 |  | -0.2811 | -0.5615 to 0.05710 | 0.0919 |  | 0.009738 | -0.3242 to 0.3415 | 0.9544 |  | 0.01514 | -0.3194 to 0.3463 | 0.9291 |
| g__Lachnospiraceae_NK4A136_group | -0.2052 | -0.5037 to 0.1371 | 0.2231 |  | -0.3736 | -0.6284 to -0.04654 | 0.0227 |  | -0.108 | -0.4256 to 0.2333 | 0.5247 |  | -0.079 | -0.4013 to 0.2607 | 0.6421 |
| g__Mitochondria | -0.2846 | -0.5641 to 0.05331 | 0.0877 |  | -0.04924 | -0.3760 to 0.2884 | 0.7723 |  | -0.001309 | -0.3340 to 0.3317 | 0.9939 |  | -0.1188 | -0.4345 to 0.2229 | 0.4836 |
| g__Parabacteroides | -0.09905 | -0.4181 to 0.2418 | 0.5597 |  | -0.2141 | -0.5106 to 0.1279 | 0.2031 |  | -0.002805 | -0.3354 to 0.3304 | 0.9869 |  | -0.06233 | -0.3872 to 0.2763 | 0.714 |
| g__Porphyromonas | 0.3489 | 0.01810 to 0.6108 | 0.0343 |  | 0.3084 | -0.02724 to 0.5816 | 0.0633 |  | 0.1161 | -0.2255 to 0.4323 | 0.4939 |  | 0.09164 | -0.2488 to 0.4120 | 0.5896 |
| g__Pseudopropionibacterium | 0.3435 | 0.01194 to 0.6069 | 0.0374 |  | 0.4954 | 0.1947 to 0.7110 | 0.0018 |  | 0.2285 | -0.1130 to 0.5217 | 0.1737 |  | 0.405 | 0.08330 to 0.6502 | 0.0129 |
| g__Romboutsia | -0.1006 | -0.4194 to 0.2404 | 0.5538 |  | -0.299 | -0.5747 to 0.03764 | 0.0722 |  | -0.09622 | -0.4158 to 0.2445 | 0.571 |  | -0.1193 | -0.4349 to 0.2225 | 0.482 |
| g__Roseburia | -0.01156 | -0.3431 to 0.3226 | 0.9459 |  | -0.319 | -0.5893 to 0.01556 | 0.0543 |  | -0.03264 | -0.3616 to 0.3035 | 0.8479 |  | 0.1008 | -0.2402 to 0.4196 | 0.5529 |
| g__Ruegeria | -0.148 | -0.4583 to 0.1945 | 0.3821 |  | -0.387 | -0.6377 to -0.06209 | 0.018 |  | -0.09281 | -0.4129 to 0.2477 | 0.5848 |  | -0.1 | -0.4190 to 0.2409 | 0.5558 |
| g__Sphingomonas | -0.2483 | -0.5368 to 0.09221 | 0.1384 |  | -0.3111 | -0.5836 to 0.02430 | 0.0609 |  | 0.005527 | -0.3280 to 0.3378 | 0.9741 |  | -0.3222 | -0.5916 to 0.01200 | 0.0518 |
| g__Subdoligranulum | -0.1692 | -0.4753 to 0.1734 | 0.3168 |  | -0.3317 | -0.5985 to 0.001358 | 0.0449 |  | -0.05168 | -0.3781 to 0.2861 | 0.7613 |  | -0.008331 | -0.3403 to 0.3255 | 0.961 |
| g__Sulfurimonas | -0.07549 | -0.3984 to 0.2640 | 0.657 |  | -0.1116 | -0.4285 to 0.2299 | 0.511 |  | 0.1017 | -0.2393 to 0.4204 | 0.5491 |  | -0.04714 | -0.3742 to 0.2903 | 0.7817 |
| g__Thiothrix | -0.09877 | -0.4179 to 0.2421 | 0.5608 |  | -0.2369 | -0.5281 to 0.1042 | 0.1581 |  | 0.06843 | -0.2706 to 0.3924 | 0.6874 |  | 0.03022 | -0.3057 to 0.3595 | 0.8591 |
| g__Veillonella | 0.3624 | 0.03356 to 0.6204 | 0.0275 |  | 0.1456 | -0.1969 to 0.4563 | 0.39 |  | -0.07754 | -0.4001 to 0.2621 | 0.6483 |  | -0.01043 | -0.3421 to 0.3236 | 0.9511 |
